# Ancient origin of the biosynthesis of lignin precursors

**DOI:** 10.1186/s13062-015-0052-y

**Published:** 2015-05-21

**Authors:** Leen Labeeuw, Patrick T Martone, Yan Boucher, Rebecca J Case

**Affiliations:** Department of Biological Sciences, University of Alberta, Edmonton, AB, T6G 2E9, , Canada; Department of Botany and Biodiversity Research Centre, University of British, Columbia, BC, V6T 1Z4, , Canada

**Keywords:** Lignin, Monolignol, Algae, Evolution, Haptophyte, Chlorophyte, Rhodophyte, Diatom, Cryptophyte, Dinoflagellate

## Abstract

**Background:**

Lignin plays an important role in plant structural support and water transport, and is considered one of the hallmarks of land plants. The recent discovery of lignin or its precursors in various algae has raised questions on the evolution of its biosynthetic pathway, which could be much more ancient than previously thought. To determine the taxonomic distribution of the lignin biosynthesis genes, we screened all publicly available genomes of algae and their closest non-photosynthetic relatives, as well as representative land plants. We also performed phylogenetic analysis of these genes to decipher the evolution and origin(s) of lignin biosynthesis.

**Results:**

Enzymes involved in making *p*-coumaryl alcohol, the simplest lignin monomer, are found in a variety of photosynthetic eukaryotes, including diatoms, dinoflagellates, haptophytes, cryptophytes as well as green and red algae. Phylogenetic analysis of these enzymes suggests that they are ancient and spread to some secondarily photosynthetic lineages when they acquired red and/or green algal endosymbionts. In some cases, one or more of these enzymes was likely acquired through lateral gene transfer (LGT) from bacteria.

**Conclusions:**

Genes associated with *p*-coumaryl alcohol biosynthesis are likely to have evolved long before the transition of photosynthetic eukaryotes to land. The original function of this lignin precursor is therefore unlikely to have been related to water transport. We suggest that it participates in the biological defense of some unicellular and multicellular algae.

**Reviewers:**

This article was reviewed by Mark Ragan, Uri Gophna, Philippe Deschamps.

## Background

Lignin is a complex and highly recalcitrant form of carbon often thought to be one of the key innovations of land plants, allowing the movement of plants from aquatic habitats to terrestrial ecosystems [1,2]. It is the second most abundant biopolymer on earth, after cellulose [3]. In land plants, lignin is deposited in the secondary cell wall and provides structural support, giving rigidity and strength to stems [4,5], as demonstrated by lignin-deficient mutants with low structural support [6,7]. The hydrophobic nature of lignin makes it impermeable to water and is thus crucial for a vascular system, enabling transport of water throughout the plant [5]. In addition, lignin has been suggested to protect plants from biological attacks. Being recalcitrant to biological degradation, increased lignification can offer protection when a plant is damaged [8]. The precursors of lignin (*p*-coumaric acid, *p*-coumaroyl-CoA, *p*-coumaraldehyde) and its monomers (monolignols *p*-coumaryl alcohol, coniferyl alcohol and sinapyl alcohol) have also been suggested to have antimicrobial properties [9,10].

**Figure 1:**
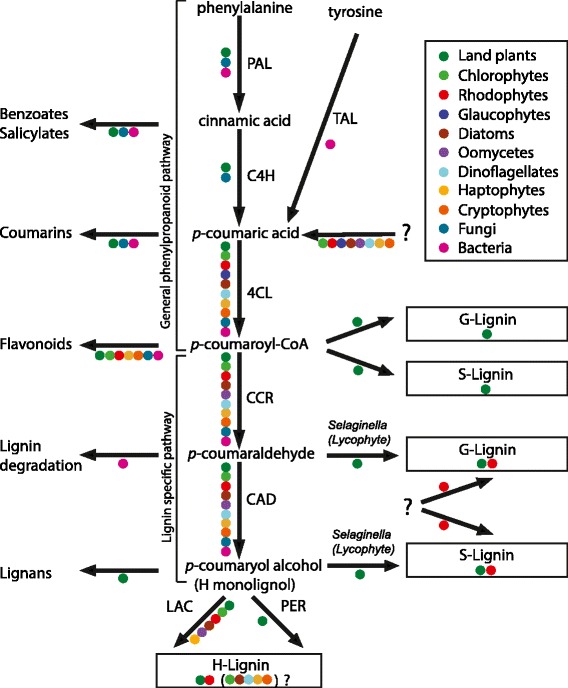
**The lignin biosynthesis pathway.** Coloured dots represent the presence of a given enzyme in a specific taxonomic group. The abbreviations used for the enzyme are: phenylalanine ammonia-lyase (PAL); cinnamate 4-hydroxylase (C4H); 4-coumarate:CoA ligase (4CL); cinnamoyl-CoA reductase (CCR); cinnamyl alcohol dehydrogenase (CAD); peroxidase (PER); and laccase (LAC). Question marks (?) indicate that the enzyme responsible for a specific function or its substrate is unknown, or that the presence of a particular compound in a given taxonomic group is hypothesized but has not been demonstrated.

Lignin biosynthesis starts with the general phenylpropanoid pathway, which can generate precursors to a diverse group of compounds including flavonoids, coumarins, quinones, and monolignols (Figure 1). The starting point of this pathway is the amino acid phenylalanine, which is deaminated to form cinnamic acid, followed by *p*-coumaric acid and *p*-coumaroyl-CoA [11]. The lignin specific pathway then uses *p*-coumaroyl-CoA to produce the simplest monolignol, H monolignol (*p*-coumaryl alcohol), through a series of reduction reactions that modify the side chain [7] (Figure 1). Production of the more structurally complex G (coniferyl alcohol) and S (sinapyl alcohol) monolignols requires additional enzymes using intermediates of H monolignol synthesis as substrates, which perform O-methylation and hydroxylation at various sites on the phenolic ring [2]. After their biosynthesis inside the cell, the monolignols are transported to the cell wall through an unknown mechanism, before undergoing cross-linking of the monomers to form the lignin polymer. This process, called lignification, is not well understood but involves simple radical coupling reactions between monolignols and a growing lignin polymer [12]. The simple coupling reactions are believed to be carried out by peroxidases and laccases, enzymes that are diverse and abundant in eukaryotes [3,13].

The lignin biosynthesis pathway has been extensively studied in vascular plants, and was thought to be a hallmark of this group [14]. Gymnosperms (softwood) are usually primarily composed of G lignin [15], angiosperm dicots (hardwood) are composed of predominantly G and S lignins while monocots (such as grasses) usually have more H lignin content [3,13]. Investigation of so called “lower” (non-vascular) plants revealed the evolution of lignin biosynthesis to be more ancient than the origin of vascular plants. *Selaginella moellendorfii* (spikemoss), which is part of the most ancient lineage of vascular plants, the lycophytes, can synthesize all three types of lignin, including the supposedly-derived S lignin, which is synthesized using an enzyme that is absent from all other land plants [16,17]. Therefore, *S. moellendorfii* likely developed this ability independently of angiosperms, which were previously the only organisms believed capable of synthesizing S lignin. Even the moss *Physcomitrella patens*, a representative of the most ancient group of land plants, the non-vascular bryophytes, encodes all lignin biosynthesis enzymes necessary to synthesize H and G lignins in its genome [4]. Although true polymerized lignin has yet to be found in this organism, it contains monolignols and lignin-like molecules, [4,18,19]. More surprising still is that H, G and S lignins have recently been identified in the cell walls of the calcified red alga *Calliarthron cheilosporioides* (Rhodophyta) [20]. Given that land plants are separated by a large evolutionary distance from rhodophytes, convergent evolution of lignin biosynthetic genes has been suggested as an explanation of how *C. cheilosporioides* acquired lignified cells [13]. There is some evidence that lignin might also be found in other algae besides rhodophytes, but it is still heavily debated. Homologs of some of the genes for lignin biosynthesis have also been found in green algae and diatom species [19]. Charophytes, a class of freshwater streptophyte algae, have been suggested to contain lignin-like compounds [21,22], as have some brown algae [23-25]. Later studies argued against this, claiming that brown algae contain only phenol compounds and not specifically lignin [12,26].

Recent research has found that the lignin precursor, *p*-coumaric acid, as well as key genes of the lignin biosynthesis pathway are present in the haptophyte *Emiliania huxleyi* [27]. Another study also found coumaric acid as well as flavonoid compounds derived from it, in a diatom (*Phaedactylum tricornutum*), a haptophyte (*Diacronema lutheri*) and several green algae (*Tetraselmis suecica*, *Chlorella vulgaris* and *Haematococcus pluviaris*) [28]. It is surprising that haptophytes and diatoms contain a lignin precursor, as well as the genes potentially encoding enzymes to convert it into monolignol. Hapthophytes and diatoms are evolutionarily very distant to plants, red and green algae and to each other. The potential for these two phylogenetically disparate phytoplanktons to synthesize monolignols suggests that the pathway for lignin synthesis might have evolved in ancient oceans and may vastly predate the origin of land plants. To investigate this possibility, we screened all available algal genomes for the presence of the lignin biosynthesis pathway, in addition to a selection of representative species of land plants and other eukaryotes belonging to various supergroups (unikonts, archaeplastids, stramenopiles, alveolates, cryptophytes, haptophytes). Although known genes required for the synthesis of G and S lignins were mostly absent outside of land plants, homologs of those needed for making the monomer of the simplest form, *p*-coumaryl alcohol (H monolignol), were found in green algae, red algae and glaucophytes, as well as multiple organisms in supergroups other than Archaeplastida. Phylogenetic analysis of the *p*-coumaryl alcohol biosynthesis genes suggests an early origin for this metabolic process, followed by at least three examples of independently evolved enzymes allowing organisms to make more complex derivatives, G and S monolignols, in red algae, club mosses and the ancestor of vascular plants.

## Results and discussion

### The lignin biosynthetic pathway has a conserved and taxonomically widespread core

An extensive screen for homologs of the known lignin biosynthesis genes was performed across all domains of life, with a specific focus on eukaryotes (Tables 1 and 2). Previous research has focused on lignin biosynthesis in *Arabidopsis* [29-32] and other model land plants [2,19,33-35], so only representative species of this group have been included in our search. As expected, all the lignin biosynthesis genes were found in all land plants for which genomes are available, with the exception of ferulate 5-hydroxylase (F5H), which is absent from the only bryophyte (moss) in our dataset, *Physcomitrella patens* [7]. Surprisingly, four gene families had a wide distribution across the various eukaryotic supergroups and were not restricted to land plants. These include: 4-coumarate:CoA ligase (4CL); cinnamoyl-CoA reductase (CCR); cinnamyl alcohol dehydrogenase (CAD); and caffeoyl-CoA *O*-methyltransferase (CCoAMT) (Tables 1 and 2).

**Table 1:** **Distribution of lignin biosynthesis genes in archaeplastid genomes**

**Organism**	**Phylum**	**PAL**	**C4H**	**4CL**	**CCR**	**CAD**	**HCT**	**C3H**	**COMT**	**CCoAMT**	**F5H**	**CSE**	***sm*** **F5H**	**PER**	**LAC**
**ARCHAEPLASTIDS**															
**Land plants**															
*Arabidopsis thaliana*	Streptophyta	+	+	+	+	+	+	+	+	+	+	+		+	+
*Oryza sativa*	Streptophyta	+	+	+	+	+	+	+	+	+	+	+		+	+
*Physcomitrella patens*	Streptophyta	+	+	+	+	+	+	+	+	+		+		+	+
*Picea abies*	Streptophyta	+	+	+	+	+	+	+	+	+	+	+		+	+
*Selaginella moellendorffii*	Streptophyta	+	+	+	+	+	+	+	+	+	+	+	+	+	+
**Green algae**															
*Bathycoccus prasinos*	Chlorophyta					+^G^				+^G^					
*Botryococcus braunii*	Chlorophyta					+^E^				+^E^					
*C. reinhardtii*	Chlorophyta				+	+				+^G^					+^G^
*Chlorella sp. NC64A*	Chlorophyta				+^G^	+^G^				+^G^					+^G^
*Chlorella variablilis*	Chlorophyta			+^G^	+^G^	+^G^				+^G^					+^G^
*Coccomyxa subellipsoidea*	Chlorophyta			+^G^	+	+				+			+^G^		+^G^
*Dunaliella salina*	Chlorophyta					+^E^									
*Haematococcus pluvialis*	Chlorophyta					+^E^									
*M. pusilla CCMP1545*	Chlorophyta					+^G^				+^G^					
*Micromonas sp. RCC299*	Chlorophyta					+^G^				+^G^					
*O. lucimarinus*	Chlorophyta					+									
*Ostreococcus sp.RCC809*	Chlorophyta					+^G^									
*Ostreococcus tauri*	Chlorophyta					+^G^				+^G^					
*Polytomella sp.*	Chlorophyta					+^G^									
*Polytomella parva*	Chlorophyta					+^E^									
*Prototheca wickerhamii*	Chlorophyta					+^E^									
*Volvox carteri*	Chlorophyta				+^G^	+				+^G^					
**Red algae**															
*C. tuberculosum*	Rhodophyta			+^G^	+^G^	+^G^				+^G^					+^G^
*Chondrus crispus*	Rhodophyta			+^G^	+^G^	+^G^									+^G^
*Cyanidioschyzon merolae*	Rhodophyta				+^G^	+^G^				+^G^					
*Galdiera sulphararia*	Rhodophyta			+^G^		+^G^									
*Porphyridium cruentum*	Rhodophyta			+^E^	+^E^	+^E^				+^E^					
**Glaucophytes**															
*Cyanophora paradoxa*	Glaucophyta			+^G^	+^G^	+^G^									

Footnote = + Present in both genome sequence and EST library, +^E^ Present in EST library only, +^G^ Present in genome sequence only. Presence (+) is determined by a reciprocal BLASTP hit with an E-value < 1x10^-30^ or less using the characterized land plant *A. thaliana* or *S. moellendorffii* gene as a query, searching the NCBI, JGI, and Congenie databases. The abbreviations used for the enzyme can be described as follows: phenylalanine ammonia-lyase (PAL); cinnamate 4-hydroxylase (C4H); 4-coumarate:CoA ligase (4CL); cinnamoyl-CoA reductase (CCR); cinnamyl alcohol dehydrogenase (CAD); *p*-hydroxycinnamoyl-CoA (HCT); *p*-coumarate 3-hydroxylase (C3H); caffeic acid O-methyltransferase (COMT); caffeoyl-CoA O-methyltransferase (CCoAMT); ferulate 5-hydroxylase (F5H); caffeoyl shikimate esterase (CSE) and *Selaginella moelledorfii* F5H (smF5H); peroxidase (PER); and laccase (LAC).

**Table 2:** **Distribution of lignin biosynthesis genes in non-archaeplastid genomes**

**Organism**	**Phylum**	**PAL**	**C4H**	**4CL**	**CCR**	**CAD**	**HCT**	**C3H**	**COMT**	**CCoAMT**	**F5H**	**CSE**	***sm*** **F5H**	**PER**	**LAC**
**STRAMENOPILES**															
**Diatoms**															
*Fragilariopsis cylindrus*	Bacillariophyta			+	+^E^	+									+
*Phaeodactylum tricornutum*	Bacillariophyta			+	+	+									+^E^
*T. pseudonana*	Bacillariophyta			+^G^											
**Pelagophyte**															
*A. anophagefferens*	Heterokontophyta			+^G^		+^G^									
**Brown algae**															
*Ectocarpus siliculosus*	Phaeophyceae			+^G^											
**Eustigmatophytes**															
*N. gaditana*	Eustigmatophyceae			+^G^	+^G^										
**Chrysophytes**															
*Ochromonas danica*	Chrysoophyceae									+^E^					
**Oomycetes**															
*Albugo laibachii*	Heterokontophyta			+^G^	+^G^	+^G^									+
*Phytophthora infestans*	Heterokontophyta			+	+	+									+
*Phytophthora sojae*	Heterokontophyta			+		+									+
**ALVEOLATES**															
**Apicomplexa**															
*Cryptosporidium muris*	Apicomplexa				+^G^	+									
*Cryptosporidium parvum*	Apicomplexa				+^G^	+									
*Theileria parva*	Apicomplexa														
*Toxoplasma gondii*	Apicomplexa														
**Dinoflagellates**															
*Symbiodinium minutum*	Dinoflagellata			+^G^	+^G^	+^G^									
**Ciliates**															
*Paramecium tetraurelia*	Ciliophora			+^G^		+									
*T. thermophila*	Ciliophora			+^G^	+^G^										
**HAPTOPHYTES**															
*E. huxleyi CCMP1516*	Haptophyta			+	+	+				+					+^G^
**CRYPTOPHYTES**															
*Guillardia theta*	Cryptophyta			+^G^	+^G^	+^G^				+^G^					
*Hemiselmis andersenii*	Cryptophyta														

Footnote = + Present in both genome sequence and EST library, +^E^ Present in EST library only, +^G^ Present in genome sequence only. Presence (+) is determined by a reciprocal BLASTP hit with an E-value < 1x10^-30^ or less using the characterized land plant *A. thaliana* or *S. moellendorffii* gene as a query, searching the NCBI, JGI, OIST Marine Genomics Unit (http://marinegenomics.oist.jp/genomes/gallery/) and *Paramecium* (http://paramecium.cgm.cnrs-gif.fr/) databases. The abbreviations used for the enzyme can be described as follows: phenylalanine ammonia-lyase (PAL); cinnamate 4-hydroxylase (C4H); 4-coumarate:CoA ligase (4CL); cinnamoyl-CoA reductase (CCR); cinnamyl alcohol dehydrogenase (CAD); *p*-hydroxycinnamoyl-CoA (HCT); *p*-coumarate 3-hydroxylase (C3H); caffeic acid O-methyltransferase (COMT); caffeoyl-CoA O-methyltransferase (CCoAMT); ferulate 5-hydroxylase (F5H); caffeoyl shikimate esterase (CSE) and *Selaginella moelledorfii* F5H (smF5H); peroxidase (PER); and laccase (LAC).

The first three of these enzymes (4CL, CCR and CAD) catalyze consecutive steps in lignin biosynthesis and are sufficient to produce *p*-coumaryl alcohol from coumaric acid (Figure 1). Homologs of all three enzymes have similar taxonomic distributions, being found mostly in marine photosynthetic algae in addition to land plants. Representatives of green algae, red algae, glaucophytes, diatoms, dinoflagellates, haptophytes and cryptophytes, as well as the non-photosynthetic oomycetes, harbor homologs of these three enzymes. Although oomycetes are non-photosynthetic, they are believed to share a photosynthetic ancestor with other stramenopiles such as brown algae and diatoms [36]. If the 4CL, CCR and CAD homologs in these diverse eukaryotes indeed catalyze the same biochemical reactions as their plant homologs, the capacity to make at least the precursor of the simplest form of lignin (H lignin) would be much more widespread than currently thought (land plants and red algae).

**Figure 2:**
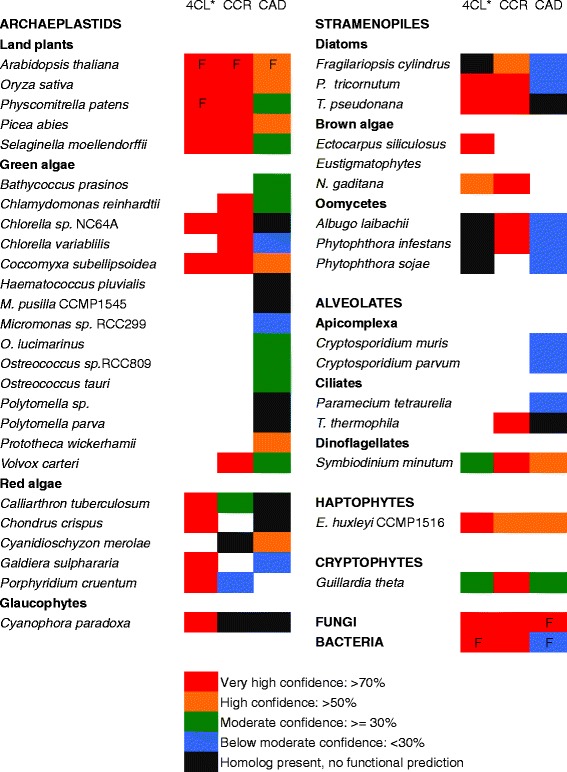
**Functional prediction of the** ***p*** **-coumaryl alcohol biosynthesis pathway genes.** Both programs used for functional prediction (Argot 2 and ESG) need to predict the correct function for it to be annotated as such. An "F" indicates that an enzyme from that taxonomic group has been biochemically characterized. No single fungi or bacteria harbour homologs for all three enzymes, but all the enzymes are found individually in some representatives of these taxonomic groups. An empty space indicates that no homolog is found in a particular species. Confidence values are derived from the ESG software. * for 4CL, as even enzymes with biochemically demonstrated function are not annotated as such with significant confidence by the softwares used, the figure indicates prediction of ligase activity.

We have performed functional prediction analysis on all homologs of 4CL, CCR and CAD homologs using the Argot2 [37] and ESG [38] packages, which are among the best performing functional annotation programs available [39] (Figure 2). Homologs of plant CCR and CAD were predicted to have a conserved function with moderate to high confidence in at least one representative of the green algae, red algae, diatoms, dinoflagellates, haptophytes and cryptophytes. For CAD, alcohol dehydrogenase function was often predicted along with cinnamyl alcohol dehydrogenase function with comparable confidence. The prediction for the specific function of 4-coumarate:CoA ligase was difficult for 4CL homologs, being weak even for enzymes that have been biochemically characterised as having that function (such as several of *Arabidopsis thaliana* paralogous 4CL enzymes) [7]. However, ligase function was predicted with high or very high confidence for all organisms with predicted CCR and CAD functions. Although homologs of all three enzymes are also found in glaucophytes and oomycetes, functional predictions were weak or absent for one or more of these enzymes in these two taxonomic groups.

Most enzymes involved in lignin biosynthesis are multifunctional or have multiple, slightly divergent, paralogous copies with different functions, either with the direction of the reaction catalyzed reversed or a change in substrate affinity. This is especially true for CAD, which catalyzes the last step in monolignol biosynthesis. SAD (sinapyl alcohol dehydrogenases) cannot be differentiated from CAD phylogenetically and both enzymes display some of the other’s specific activity [40,41]. Also, there are many enzymes with CAD activity (oxidation of an aldehyde to an alcohol) as well as alcohol dehydrogenase activity (reduction of an alcohol to an aldehyde), such as yeast ADH6 and ADH7 enzymes [42]. Given the functional flexibility in these protein families, functional switches are likely to have occurred frequently throughout eukaryotic evolution, making exact functional predictions difficult without biochemical data.

### *p*-coumaryl alcohol synthesis is likely common in photosynthetic eukaryotes

Homologs for all genes of the 4CL-CCR-CAD pathway, responsible for the synthesis of *p*-coumaryl alcohol from *p*-coumaric acid, occur in several widely divergent eukaryotic taxonomic groups (stramenopiles, haptophytes, cryptophytes, dinoflagellates and archaeplastids). This does not agree with a previously proposed origin in early land plants [3]. Several alternative hypotheses could explain this distribution. The genes of this pathway (which will be called the *p*-coumaryl alcohol biosynthesis pathway henceforth) could have been present in eukaryotic ancestors predating the origin of some or all of these groups, and lost in lineages in which the pathway is absent. The superfamilies to which 4CL (adenylate-forming enzymes), CCR (adenylate reductase) and CAD (medium-chain dehydrogenase/reductases) belong are most certainly ancestral to eukaryotes, being widespread in all eukaryotic supergroups [35,43-47]. However, close homologs to characterized plant 4CL, CCR and CAD enzymes with a correctly predicted function are not as ubiquitous. The complete *p*-coumaryl alcohol biosynthesis pathway is present almost solely in photosynthetic eukaryotes, making a scenario in which they evolve in an ancient eukaryotic ancestor and are subsequently selectively lost in non-photosynthetic taxonomic groups unlikely. A more parsimonious explanation could be that this pathway would have originated in an ancestor of green and red algae and was subsequently transferred to other taxonomic groups in the same way they acquired the capacity to photosynthesize: through taking up a red or green alga in a secondary endosymbiotic event (endosymbiotic gene transfer or EGT) [36]. Another possibility is that several genes in this pathway were acquired independently by lateral gene transfer (LGT) from bacteria or heterotrophic eukaryotes in various photosynthetic lineages. Phylogenetic analysis of the 4CL, CCR and CAD enzymes were performed to differentiate between these hypotheses on the origin(s) of the *p*-coumaryl alcohol biosynthesis pathway.

### *p*-coumaryl alcohol biosynthesis could have originated in an ancient archaeplastid

**Figure 3:**
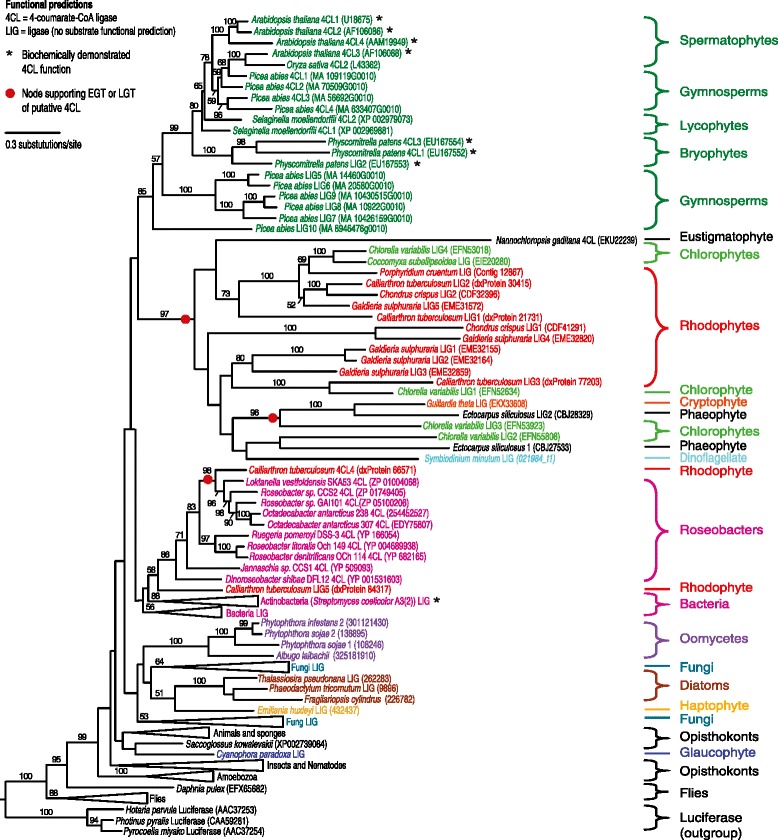
**Maximum likelihood phylogeny of the 4-coumarate:CoA ligase (4CL) enzyme from the** ***p*** **-coumaryl alcohol biosynthesis pathway.** Amino acid sequences were aligned with MUSCLE and the tree compiled using RaxML. Numbers above branches refer to bootstrap values above 50%. Luciferase, a 4CL homolog [79], was used as the outgroup. Gene names are included next to taxa when function could be predicted. * indicates that the enzyme has been biochemically characterized in this organism [29,72,73].

**Figure 4:**
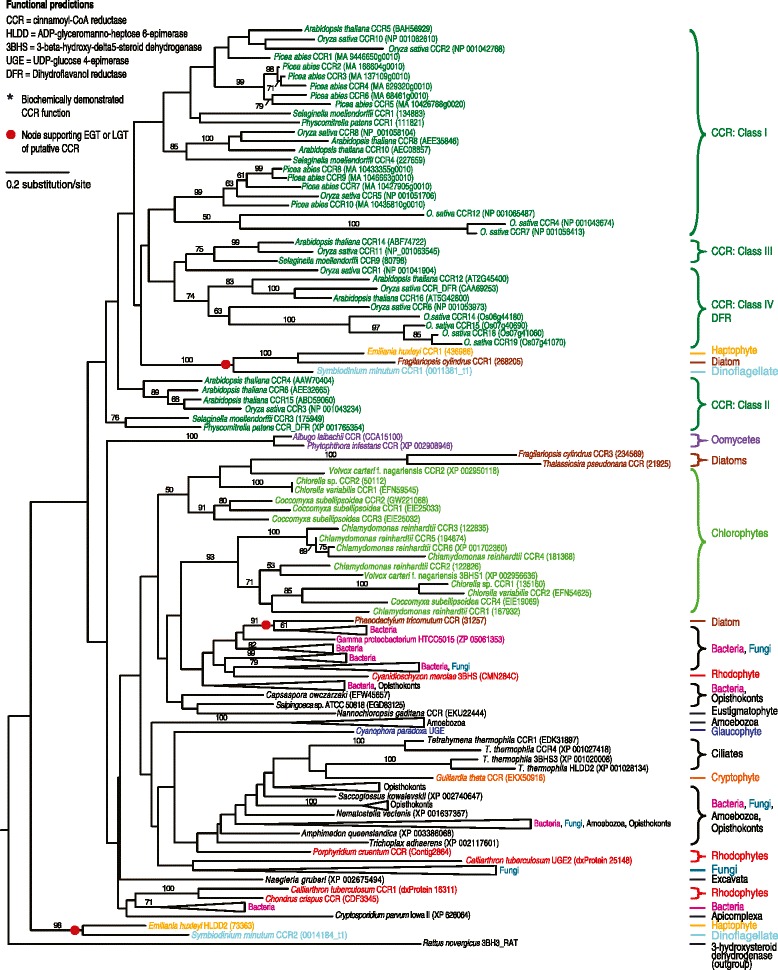
**Maximum likelihood tree of cinnamoyl-CoA reductase (CCR) enzyme from the** ***p*** **-coumaryl alcohol biosynthesis pathway.** Amino acid sequences were aligned with MUSCLE and the tree compiled using RaxML. Numbers above branches refer to bootstrap values above 50%. 3-hydroxysteroid dehydrogenase, a CCR homolog [63], was used as the outgroup. The various classes of CCR are shown based on previous research including bona fide CCR compared to CCR or CCR-like genes. In addition, genes showing high similarity to dihydroflavonol reductase (DFR) genes were included [19,45]. Gene names are included next to taxa when function could be predicted. * indicates that the enzyme has been biochemically characterized in this organism [30,69,70].

**Figure 5:**
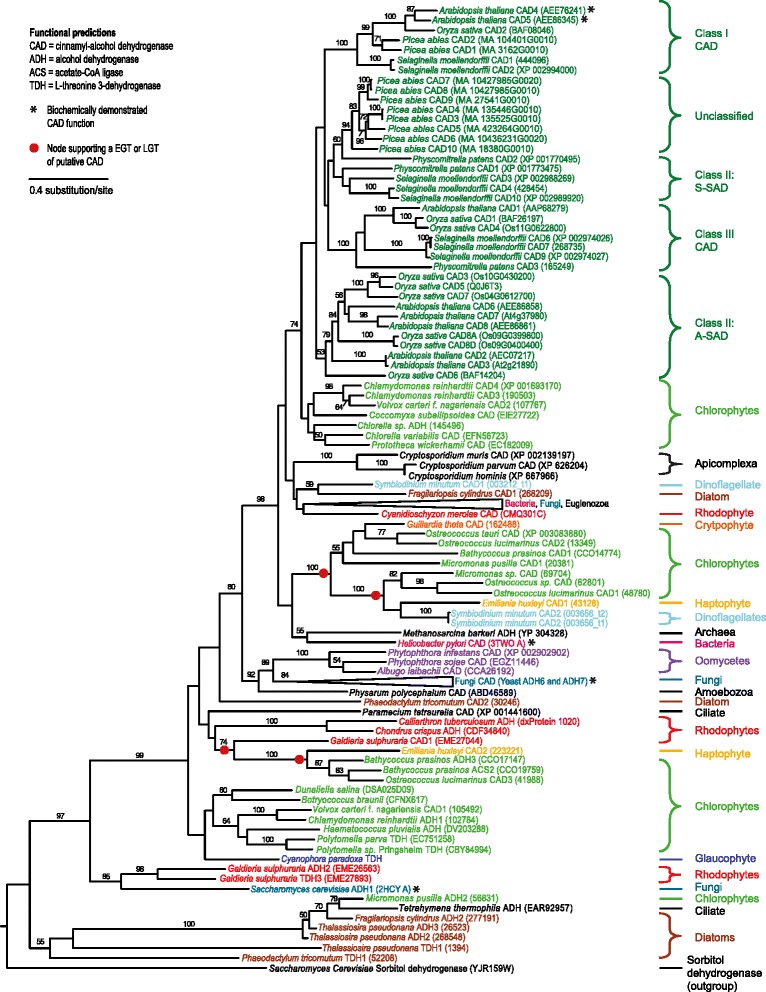
**Maximum likelihood tree of cinnamyl alcohol dehydrogenase (CAD) enzyme from the** ***p*** **-coumaryl alcohol biosynthesis pathway.** Amino acid sequences were aligned with MUSCLE and the tree compiled using RaxML. Numbers above branches refer to bootstrap values above 50%. Sorbitol dehydrogenase, a CAD homolog [80], was used as the outgroup. The various classes of CAD are shown based on previous research, including the sinapyl alchohol dehydrogenases (SAD), which share some specific activity with CAD [40,41]. Gene names are included next to taxa when function could be predicted. * indicates that the gene has been biochemically characterized in this organism [31,42,71,74].

The *p*-coumaryl alcohol biosynthesis pathway seems to be ancestral to green algae, but with frequent loss of this metabolic function throughout this taxonomic group (Figure 2). All green algal species are found within a single well-supported (97%) clade in the 4CL tree (mixed with red algae and secondarily photosynthetic organisms) (Figure 3) and in two clades in the CCR tree and three clades in the CAD tree (Figure 4 and 5). The multiple green algal clades in the CAD tree likely represent conservation of a different paralog in two major green algal lineages; prasinophytes and the core chlorophytes [48]. The core chlorophytes are monophyletic and group with land plants, while the prasinophytes are divided in two clades, the result of multiple divergent paralogs being present in *Bathycoccus prasinos* and *Ostreococcus lucimarinus.* In the CCR tree, the two green algal clades are both composed of core chlorophytes, as well as overlapping in their species content and are proximal to each other. They likely represent paralogy originating after the divergence of core chlorophytes from ancestral green algae. Despite paralogy being observed in green algae for all three genes, the presence of *p*-coumaryl alcohol biosynthesis gene homologs in various species from two major green algal groups suggests the presence of this pathway is ancestral. Green algae are never part of clades containing organisms from other taxonomic groups besides secondarily photosynthetic species and red algae, suggesting the absence of LGT for *p*-coumaryl alcohol biosynthesis gene homologs in this lineage. The fact that only two of fifteen green algal genomes or EST libraries screened contain homologs to all three *p*-coumaryl alcohol biosynthesis enzymes suggests this function has been frequently lost and is likely non-essential, as compared to its crucial role in most land plants.

The story is different for red algae. In this group, only 4CL seems to be ancestral, being found in a single clade, which also contains green algae and secondarily photosynthetic organisms (Figure 3). The only exceptions to this are two of the five 4CL paralogs in *Calliarthron tuberculosum* clustering in bacterial clades, one of them very strongly with a marine α-proteobacteria group known as the roseobacter clade, indicating a likely LGT from bacteria. For both CCR and CAD, red algae are polyphyletic, with some paralogs weakly clustering with homologs from bacteria or heterotrophic eukaryotes. It is therefore difficult to say whether the pathway was ancestrally found in red algae or some of its enzymes were acquired by LGT. Like in green algae, only a small proportion of red algal genomes investigated have homologs for all three enzymes (one in five). Although the fact that the red alga *Calliarthron cheilosporioides,* a close relative to *C. tuberculosum,* can synthesize *p*-coumaryl alcohol is well established [20], this function does not seem to be an essential feature of red algae, making the loss of it likely. This is perhaps visible in the specialized role of lignin in the uncommon lignified joints, genicula, of *C. cheilosporioides* [20].

**Figure 6:**
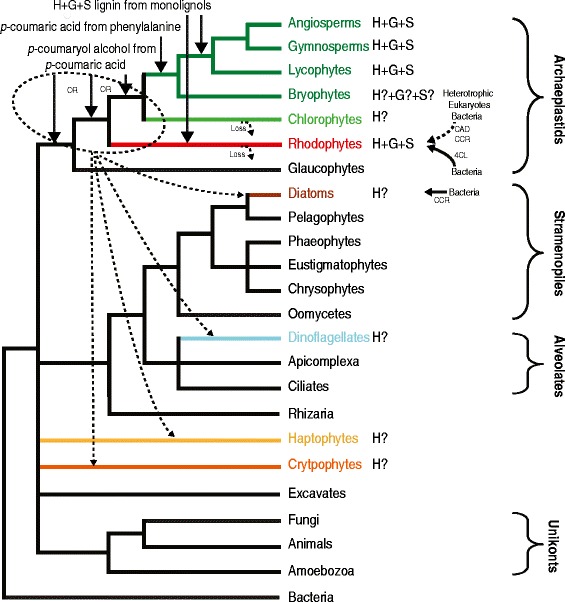
**Major evolutionary events hypothesized in the evolution of the lignin biosynthetic pathway across the eukaryotic tree.** The tree is a consensus of current phylogenetic analyses of the eukarotic domain [1,36,49]. Major events indicated by labeled arrows on the tree are hypothesized from our genome survey and phylogenetic analyses of the putative *p*-coumaryl alcohol biosynthesis enzymes. Taxonomic groups in which three or more enzymes catalyzing consecutive steps in the lignin biosynthesis pathway were found are colored. The chemical detection of polymerized lignin is indicated in the margin for each taxonomic group, with the type of lignin (either H, G or S) specified. A question mark (?) indicates that some putative lignin biosynthetic enzymes were found and fuctionally predicted but that there is currently no biochemical evidence of polymerized lignin. Arrows indicate the origin and direction of putative EGT and LGT events (solid arrows are used for events that are conclusive, dashed arrows when events are hypothesized).

These results suggest that all three genes of the *p*-coumaryl alcohol biosynthesis pathway are likely to have been present in at least the shared ancestor of plants and green algae, but possibly earlier, before the speciation of the red algal ancestor (Figure 6). A few additional features of the phylogenies support an earlier origin than the ancestor of land plants. The CAD homologs found in core chlorophytes cluster with land plants (albeit with modest 74% bootstrap support). Also, although both the CCR and CAD phylogenies likely contain hidden ancestral paralogy and differential loss, this is not the case for the 4CL tree. Paralogy is evident only for red and green algae and confined to a single clade (with the exception of copies likely acquired from bacteria by *C. tuberculosum*). As red and green algal species are polyphyletic inside this clade, duplication(s) of the 4CL homolog likely occurred in their ancestor and therefore would predate these lineages. It is difficult to say if the pathway could have been present in the archaeplastid ancestor itself, before divergence of the glaucophytes. Glaucophyte is the earliest branching lineage in archaeplastids [49] and could potentially be quite informative on the origin of the *p*-coumaryl alcohol biosynthesis pathway in this group. Unfortunately, although it harbours homologs for all three enzymes of the pathway, none of them has strong functional prediction and their position in phylogenies is unresolved. It is therefore not possible to determine if the pathway is ancestral to archaeplastids with information currently available.

### The *p*-coumaryl alcohol biosynthesis pathway likely spread through endosymbiotic gene transfer

Four very disparate groups of secondarily photosynthetic organisms have at least one representative with all three enzymes of the *p*-coumaryl alcohol biosynthesis pathway with the correct functional predictions: dinoflagellates, diatoms, hapthophytes and cryptophytes. The large majority of 4CL, CCR and CAD homologs found in these organisms cluster with each other (despite being from widely divergent taxonomic groups) or with red or green algae.

There is no observed paralogy of the 4CL gene in secondarily photosynthetic organisms. Diatoms and the haptophyte weakly cluster together (51%), the cryptophyte clusters strongly (100%) with another secondarily photosynthetic organism (*Ectocarpus silicosis*, a brown algae) and with green algae, while the dinoflagellate is found inside the same well-supported (97%) mixed green and red algal clade. For CCR, the haptophyte has two paralogs, one clustering strongly with the dinoflagellate (98%) and the other with both the dinoflagellate and the diatom *Fragilariopsis cylindrus* (100% support). Other diatom CCR paralogs weakly cluster with green algae (50% support), while the single CCR homolog found in the diatom *Phaeodactylum tricornutum* strongly groups with bacteria (91%) and was likely acquired from a member of this domain by LGT. The cryptophyte single CCR homolog position in the tree is unresolved. The CAD homologs of secondarily photosynthetic organisms show a similar pattern to 4CL and CCR. A dinoflagellate and a hapthophyte paralog strongly cluster together within a green algal clade that also includes the cryptophyte (100% support). The second dinoflagellate CAD paralog weakly clusters with the diatom *F. cylindrus* single CAD homolog (59% support) and the second haptophyte paralog groups strongly with green algae (100%).

The underlying pattern is clear: a recurring clustering of secondarily photosynthetic organisms from disparate taxonomic groups with each other or green or red algae. Although the exact pattern of species clustering varies between phylogenies (a likely result of ancient paralogy and differential loss), it suggests that most 4CL, CCR and CAD homologs present in secondarily photosynthetic species have been acquired by EGT from a red or green algae or their ancestors. This also implies that the *p*-coumaryl alcohol biosynthesis pathway, or at the very least its component genes, are ancient, predating the diversification of various major eukaryotic taxonomic groups such as the dinoflagellates, hapthophytes, cryptophytes and diatoms. Although ancient paralogy coupled with differential loss can often make phylogenies misleading, it is very unlikely that it would result in similar patterns of taxonomically unrelated secondarily photosynthetic organisms clustering with each other or with green/red algae for three different genes. The recurrent co-clustering of dinoflagellates, hapthophytes and diatoms in 4CL, CCR and CAD phylogenies is more parsimoniously explained by a common origin. The cryptophyte, *Gulliardia theta,* on the other hand, does not cluster directly with these other secondarily photosynthetic organisms in any phylogeny, suggesting an independent origin. More evidence is needed to confirm the exact origin of these genes and the number of events in which they might have been acquired by secondarily photosynthetic organisms.

### LGT could have impacted the evolution of lignin precursors biosynthesis

Previous studies have suggested that at least one gene in the lignin biosynthesis pathway, phenylalanine ammonia lyase (PAL) (Figure 1), was likely acquired through LGT from soil bacteria to an ancestor of land plants [50]. LGT is likely to also have influenced the evolution of *p*-coumaryl alcohol biosynthesis. Some of the 4CL homologs present in the red alga *C. tuberculosum* might have been affected by this phenomenon. Indeed, the 4CL phylogeny contains a strongly supported clade composed of the red alga *C. tuberculosum* grouping with bacteria, including many sequences derived from roseobacters (Figure 3). This suggests a horizontal 4CL gene transfer from a roseobacter to a red alga (Figure 3), giving *C. tuberculosum* extra copies of 4CL in addition to those it likely inherited from archaeplastid ancestors. Close physical associations have been shown to promote LGT, and two roseobacter species are known to live intracellularly and intercellularly within red macroalgae [51,52]. Some red algal species even depend on bacteria for growth or morphogenesis, highlighting the intimacy of this relationship [53,54]. This bacteria-red algae clade in the 4CL tree also includes a functionally characterized gene from the bacterium *Streptomyces coelicolor*, which has been shown to have 4CL activity [55]. Argot2 and ESG also predict all roseobacter and *C. tuberculosum* 4CL homologs in this clade to have 4CL function, a prediction only made for these bacteria, red alga and some land plants enzymes in our datasets. This makes it likely that these laterally transferred homologs have true 4-coumarate:CoA ligase function. For a LGT event from a bacterium to a eukaryote to be confirmed, bacterial genes need to be found inserted next to genuine eukaryotic genes. Unfortunately, the *C. tuberculosum* 4CL homologs are found on very small contigs in the alga’s genome and further upstream and downstream sequence data would be needed to determine if they have a bacterial or algal context. It is therefore not possible to exclude that the *C. tuberculosum* 4CL genes clustering with roseobacters represent bacterial contamination present in its genome sequence, despite systematic screening of sequence data to remove it [56]. Regardless of whether LGT between roseobacters and *C. tuberculosum* has taken place, the presence of 4CL in these bacteria raises the possibility that they could provide intermediates for the production of *p*-coumaryl alcohol to their algal host. If roseobacters can make *p*-coumaric acid (none of the known genes for doing so have yet been found in this group), the presence of 4CL would theoretically enable them to produce *p*-coumaroyl-CoA and potentially provide it to their algal host.

Another likely case of LGT is the acquisition of a putative CCR (with very high confidence functional prediction) by the diatom *P. tricornutum* from bacteria. The former is found nested inside a bacterial clade with strong support (91%). As other diatoms’ putative CCRs cluster with green algae, it is likely that a bacterial CCR displaced the homolog from algal origin previously present in *P. tricornutum*. Although LGT did not bring a novel gene to either *C. tuberculosum* or *P. tricornutum* (both had an existing homolog prior to LGT which was either displaced or complemented), it likely had an effect on their secondary metabolism.

### The phenylpropanoid pathway is unique to land plants and fungi

The phenylpropanoid pathway enzymes responsible for the production of *p*-coumaric acid from the amino acid phenylalanine, PAL and cinnamate 4-hydroxylase (C4H), were only found in land plants and fungi and are clearly missing from all other eukaryotic genomes screened (Tables 1 and 2, Figure 1). How is it possible for organisms to synthesize monolignols without these enzymes? The red alga *Calliarthron* can produce all types of lignin [20] and we could not find these enzymes encoded in the *C. tuberculosum* genome sequence (Table 1). The product made by PAL and C4H from phenylalanine, *p*-coumaric acid, has been found in an axenic culture of the haptophyte *E. huxleyi* [27], which also lacks PAL and C4H (Table 2). Since both *Calliarthron* and *E. huxleyi* have 4CL, CCR and CAD homologs, there must be other, yet to be described, enzyme(s) capable of synthesizing *p*-coumaric acid and provide it as a substrate to the 4CL-CCR-CAD pathway to produce *p*-coumaryl alcohol. Furthermore, we could not find PAL or C4H genes in the genomes of any green alga or diatom (Tables 1 and 2), although *p*-coumaric acid has been found in species from both of these groups [28].

PAL was likely acquired from bacteria by the ancestor of land plants or fungi and later horizontally transferred between these two groups [50]. C4H is also uniquely found in land plants and fungi, with distant bacterial homologs (data not shown). The combination of these two genes is therefore likely a late invention/acquisition of land plants and fungi. Land plants added two genes (C4H and PAL) to the 4CL, CCR and CAD already present in their ancestor (Figure 6). Whether PAL and C4H displaced an ancestral enzyme(s) synthesizing *p*-coumaric acid or those enzyme(s) are still present in land plant genomes is currently unknown. The only other enzyme known to synthesize *p*-coumaric acid is tyrosine ammonia lyase (TAL), which has only been found in a few bacteria [50]. It can by itself convert the amino acid tyrosine to coumaric acid, suggesting that a single unknown enzyme could carry the same function in eukaryotes lacking PAL and C4H but which have 4CL, CCR and CAD, such as the hapthophyte *E. huxleyi* and the red alga *Calliarthron.* It is also possible that enzymes analogous to PAL and/or C4H exist, as PAL activity has been found in the green alga *Chlorella pyrenoidosa* [57], but we could not find PAL homologous to plant enzymes in any of the *Chlorella* genomes screened (Table 1).

### Expansion of the lignin biosynthesis pathway has occurred multiple times independently on land and in the sea

Screening of eukaryotic genomes revealed that except for 4CL, CCR and CAD, all other lignin biosynthesis genes found in land plants are missing from *Calliarthron* (Table 1), despite the clear presence of all three lignin types in this red alga [20]. Assuming the capacity to produce 4CL’s substrate *p*-coumaric acid, the presence of 4CL, CCR and CAD genes theoretically enables the synthesis of *p*-coumaryl alcohol as well as its intermediates, which can be used as substrates for the synthesis of G and S lignins (Figure 1). This makes convergent evolution of the ability to synthesize G and S lignins in *Calliarthron* simple, as it would only require the addition of two more enzymes to this core pathway. For example, the lycophyte *S. moellendorfii* only needed to add caffeic acid *O*-methyltransferase (COMT) and ferulate 5-hydroxylase (*sm*F5H) to the enzymes needed for *p*-coumaryl alcohol synthesis to be able to make both G and S lignins [17]. Also, not all land plants can make S lignin, and the presence of both producers and non-producers in various groups of plants suggests that this ability has been gained and lost multiple times in land plants. For example, most gymnosperms do not produce S lignin, but some can, such as *Ginkgo biloba* (maidenhair tree) [15]. The fact that modifications of lignin production can easily evolve from a genetic background found in various photosynthetic eukaryotic lineages lends insight into how the red alga *Calliarthron* likely evolved the ability to produce G and S lignins. Whether this type of convergent evolution has also happened in other lineages is an open question. Provisional biochemical evidence for the presence of *p*-coumaryl alcohol in brown and green algae, but absence of G and S variants [4] suggests that numerous eukaryotes could have the capacity to only synthesize *p*-coumaryl alcohol or H lignin.

## Conclusions

The widespread distribution of coumaryol biosynthesis gene homologs across various eukaryotic supergroups suggests an ancient origin for this pathway. Although we cannot dismiss the possibility of its presence in an ancient eukaryotic ancestor and subsequent loss in all lineages in which it is absent, an origin in archaeplastids is more parsimonious. The ancient pathway for *p*-coumaryl alcohol synthesis should contain one or more gene(s) that precede 4CL, CCR and CAD, as it requires a source of *p*-coumaric acid, but these have yet to be discovered. Since *p*-coumaric acid is found in the haptophytes *E. huxleyi* and *D. lutheri*, the diatom *P. tricornutum* and the green alga *C. vulgaris* [27,28], and none of these lineages contains any homologs of PAL and C4H, there is little doubt in the existence of enzyme(s) with an analogous function(s). It is not implied that any organisms carrying this ancient pathway can necessarily polymerize *p*-coumaryl alcohol (H monolignol) to form H lignin or even synthesize the more complex G and S monolignols, but they are likely able to synthesize at least *p*-coumaryl alcohol. As all the secondary photosynthetic organisms investigated as well as most green and red algae are marine organisms, it is intriguing to consider an authentic marine source of monolignols or lignin. As these compounds and their degradation products are used as biomarkers to calibrate for terrestrial carbon input into marine systems [58,59], marine sources of monolignols or lignin therefore have the potential to redefine our understanding of the marine carbon cycle.

The function of *p*-coumaryl alcohol in unicellular marine photosynthetic eukaryotes such as diatoms, dinoflagellates, hapthophytes, cryptophytes and some green and red algae, is unclear. The two main roles of lignins derived from *p*-coumaryl alcohol and other monolignols in land plants are water transport and structural support. Water transport systems are absent in unicellular algae. If the *p*-coumaryl alcohol likely produced by unicellular and photosynthetic eukaryotes is polymerized as lignin or lignans, it could also contribute to their structural strength, although other compounds such as silica and cellulose are already known fulfil this function in such organisms [60]. More likely functions that can be fulfilled by *p*-coumaryl alcohol are UV protection and microbial defence. Phenolic compounds such as *p*-coumaric acid and its derivatives exhibit high UV absorptivity and could potentially protect an organism against the damaging effects of sunlight [61]. The lignin biosynthetic pathway has also been implicated in the defence system of plants, as individual enzymes (e.g., CAD, CCR and CCoAMT) have been shown to defend against microbial attacks [8,9,62,63]. Intermediates of the lignin biosynthesis pathway have also been shown to have antimicrobial properties [10,64,65]. Such a role in host defense or UV protection may have provided selection for the early evolution of lignin biosynthetic pathway in the ocean, which was then co-opted for water transport and structural strength in land plants faced with new selective pressures of an air-land environment.

## Methods

### Database search

Sequences for the enzymes in the phenylpropanoid and lignin specific pathways of *Arabidopsis thaliana* were found in the *Kyoto Encyclopedia of Genes and Genomes (*KEGG) database (http://www.genome.jp/kegg/), as well as previous literature [19,45,66]. Additional enzymes from the lignin pathway found only in *Selaginella moellendorffii* were obtained from the KEGG database and previous literature [16]*.* Whenever possible, proteins that have been biochemically characterized were used as queries. These protein sequences [NCBI: NP_179765, NCBI: NP_188576, NCBI: NP_173872, NCBI: NP_001077697, NCBI: NP_173047, NCBI: NP_181241, NCBI: NP_180607, NCBI: NP_199704, NCBI: NP_200227, NCBI: NP_850337, NCBI: NP_195345, NCBI: XP_002963471, NCBI: AAB09228, NCBI: XP_002992167] were used to query the National Centre for Biotechnology Information (NCBI) database (http://www.ncbi.nlm.nih.gov/) for homologs, using BLASTp and tBLASTn searches of the protein, genome and expressed sequence tags (EST) databases. An e-value of 10^-30^ or less was used as a stringent cut-off for homology. A reciprocal BLASTp search of the hits on *Arabidopsis thaliana* proteins was then performed to confirm orthology. Searches of the algal genomes were carried out using the Department of Energy (DoE) Joint Genome Institute (JGI) database (http://genome.jgi.doe.gov) using the latest releases as of April 2014 of the dataset created with ‘all models’ gene prediction algorithms. The algal genomes included were: *Chamydomonas reinhardtii, Chlorella sp.* NC64A*, Micromonas sp.* RCC299*, Micromonas pusilla CCMP 1545, Ostreococcus lucimanarinus, Ostreococcus sp.* RCC809*, Volvox carteri f. nagariensis, Fragilariopsis cylindrus, Thalassiosira pseudonana, Phaeodactylum tricornutum, Aureococcus anophagefferens,* and *Emiliania huxleyi* CCMP 1516. Additional algal genomes were retrieved from NCBI, including: *Bathycoccus prasinos, Botryococcus braunii, Chlorella variablilis, Coccomyxa subellipsoidea, Dunaliella salina, Haematococcus pluvialis, Ostreococcus lucimarinus, Ostreococcus tauri, Polytomella sp., Polytomella parva, Prototheca wickerhamii, Chondrus crispus, Galdiera sulphararia, Ectocarpus siliculosus, Nannochloropsis gaditana, Ochromonas danica, Guillardia theta,* and *Hemiselmis andersenii.* The genome of the dinoflagellate *Symbiodinium minutum* was searched using http://marinegenomics.oist.jp. Additionally, the genomes of the following reference land plants were searched in NCBI: *Oryza sativa, Physcomitrella patens, Selaginella moellendorffii.* The genomes from non-photosynthetic species closely related to chloroplast-bearing taxa were also specifically searched: *Albugo laibachii, Phytophthora infestans, Phytophthora sojae, Cryptosporidium muris, Cryptosporidium parvum, Theileria parva, Toxoplasma gondii,* and *Tetrahymena thermophile. Paramecium tetraurelia*, using http://paramecium.cgm.cnrs-gif.fr/db/tool and *Picea abies* using http://congenie.org/blastsearch, were also searched. Local searches against red algal genomes were carried out for *Calliarthron tuberculosum, Cyanidioschyzon merolae,* and *Porphyridium cruentum* [56]. *C. tuberculosum* is very closely related to the other species of *Calliarthron*, *C. cheilosporioides*, in which lignin was physically identified [67]. In addition, Dr Adrian Reyes performed a local search on *Cyanophora paradoxa* genome [68].

### Functional prediction

Functional prediction was performed on all homologs of enzymes in the phenylpropanoid and lignin specific pathways found in our public database searches. Two protein function prediction packages were used, Argot2 [37] as well as ESG [38]. These are two of the top programs for protein function prediction according to the ongoing Critical Assessment of protein Function Annotation (CAFA) study [39]. Argot2 performs BLAST and HMMer searches of sequence databases and then annotates the results with GO (Gene Ontology) terms retrieved from the UniProtKB-GOA database and terms which are then weighted using the e-values from BLAST and HMMer. The weighted GO terms, which can also be provided directly, are processed according to both their semantic similarity relations described by the Gene Ontology and their associated score. ESG recursively performs PSI-BLAST searches from sequence hits obtained in the initial search from the target sequence, thereby performing multi-level exploration of the sequence similarity space around the target protein. Each sequence hit in a search is assigned a weight that is computed as the proportion of the log(E-value) of the sequence relative to the sum of log(E-value) from all the sequence hits considered in the search of the same level, and this weight is assigned for GO terms annotating the sequence hit. The weights for GO terms found in the second level search are computed in the same fashion. Ultimately, the score for a GO term is computed as the total weight from the two levels of the searches. The score for each GO term ranges from 0 to 1.0.

### Phylogenetic analysis

As only land plants contained most genes found in the phenylpropanoid and lignin biosynthesis pathways, only the three core enzymes of the *p*-coumaryl alcohol biosynthesis pathway were further analyzed: cinnamyl alcohol dehydrogenase (CAD), 4-coumarate:CoA ligase (4CL), and cinnamoyl-CoA reductase (CCR). In order to ensure the sequence dataset was complete, additional sequences of enzymes from reference plant genomes characterized in previous literature were added to the datasets [19,29-31,40-42,45,69-74]. Protein sequences were imported into Geneious Pro v5.5 (Biomatters, New Zealand) [75] and multiple alignments were constructed using MUSCLE [76]. The alignments were then edited in Geneious. Core functional domains and motifs were determined using the NCBI conserved domain search on the *Arabidopsis thaliana* proteins as well as previous literature [40,77]. Proteins in which these core motifs were not conserved were eliminated. Poorly aligned regions were manually edited. The alignments were then imported into Randomized Axelerated Maximum Likelihood v.7.0.4 (RAxML) [78] (http://sco.h-its.org/exelixis/software.html) to create a maximum likelihood phylogenetic trees (WAG substitution model, 100 bootstrap replicates, gamma distribution parameter estimated). The tree was formatted for presentation in FigTree v.1.3 (http://tree.bio.ed.ac.uk/software/figtree/).

## Reviewers’ comments

### Reviewer’s report 1

#### Mark ragan

The authors present computationally based evidence that three genes (4CL, CCR and CAD) likely to encode the successive enzymatic steps that reduce the side chain of *p*-coumaric acid to the corresponding alcohol are present in diverse (mostly photosynthetic) protists, and in red and green algae. On the other hand, genes encoding the biosynthesis of p-coumarate from phenylalanine are known only from land plants and fungi. The authors reasonably suggest a scenario in which capability for stepwise reduction of p-coumarate was transferred by secondary endosymbioses from an ancient red or green alga to other lineages. Conservative settings are used for sequence matching, and in general the results are interpreted with adequate caution.

Despite the title and use of prejudicial terminology (monolignol) throughout the article, no new evidence is presented that lignin is actually produced by organisms other than green plants, green algae and the red alga *Calliarthron*. The seeming coding capacity to synthesize p-coumarate and (in some diatoms, oomycetes and *Emiliania*) laccase is certainly suggestive, and the authors point out potential functions of monolignols other than in generation of a cross-linked structural polymer. Nonetheless it remains easy for the reader to over-interpret the results. I urge the authors to be much more sparing in their usage of the term monolignol.

*AU: We agree with the reviewer that the term monolignol can be prejudicial. We have replaced it with p-coumaryl alcohol wherever possible. p-coumaryl alcohol and H monolignol are essentially the same chemical compound, but the former name does not implies that it is used to make lignin. We have also changed the title to make it less prejudicial, simply referring to synthesis of lignin precursors instead of lignin.*

Just as CAD not clustering directly with land plants enzymes does not necessarily mean that homologs from other organisms have a different function, the inferred homology of CAD between green algae and plants does not require shared derived function.

*AU: We agree that the clustering of green algae CAD with land plants is only suggestive of shared function and does not demonstrate it. We have changed the text to remove implications of shared function based on phylogenetic clustering.*

Although the authors use a conservative BLAST threshold, require reciprocal best hits (RBHs) and where possible query with sequences from biochemically characterised proteins, they do not mobilise further evidence (e.g. shared domain structure) potentially supportive of shared function, and rightly call attention to paralogy and functional overlap among some lignin-biosynthetic genes in green plants. RBH does not confirm orthology (Methods, paragraph 1); it would be better to refer to database match partners simply as such, or if needs be as putative homologs.

*AU: We have changed our use of terminology to avoid implying orthology when it has not been clearly demonstrated. We have also performed additional functional analysis on all proteins of interest using the programs Argot2* [37] *as well as ESG* [38]*, two of the top programs for protein function prediction according to the CAFA study* [39] *and included the results in Figure**2**. We have also removed from the phylogenies all proteins without the correct function predicted.*

Regarding the title: the authors do not build a case for multiple origins, nor that this origin (or origins) occurred in the ocean.

*AU: We have changed the title to remove reference to multiple origins and the ocean.*

### Reviewer’s report 2

#### Uri gophna

The authors present an interesting and comprehensive analysis of the evolution of lignin biosynthesis, and infer an ancient origin for it in the ancestor of archaeplastids. The topic is of high interest and the phylogenetic analysis is robust. I have two comments:

The authors elaborate on lignin biosynthesis in the red alga *Calliarthron* that has a 4 CL homolog that clusters orthologs from bacteria of the roseobacter clade. While it is tempting to suggest LGT from these bacteria, that are often algal symbionts, the authors acknowledge that these homologs can be also " represent bacterial contamination present in its genome sequence". Regardless of whether the gene has stably integrated into the algal genome or its enzyme product is obtained from the symbionts by the alga without gene transfer, association with these bacteria could contribute to the lignin synthesis of the eukaryote over evolutionary timescales. Additionally, has the role of this pathway been investigated in the roseobacter clade, and has it been connected to pigment production in these marine bacteria that are often host-associated?

*AU: Although there is no current evidence of bacterial symbionts of algae providing them with metabolic precursors for synthesis of bioactives or structural components, we acknowledge the possibility of such a situation. After screening all currently available roseobacter genomes for lignin biosynthesis genes, we have only found 4CL is some species, while CCR and CAD were absent. We cannot rule out that a yet to be identified roseobacter symbiont of algae would have these two genes, as putative homologs have been found in some bacteria. If roseobacters can make p-coumaric acid (none of the known genes for doing so have been found in this group), the presence of 4CL would theoretically enable roseobacters to produce p-coumaroyl-CoA and potentially provide it to their algal host. We now mention this possibility in the text.*

The authors conclude that "Monolignols and their polymerized forms, such as lignin, are also likely to have originally had a different function than structural support or water transport". While there are many functions, such as antibacterial activity, for intermediates in the pathway, polymerized forms such as lignin are very hydrophobic and resistant. Thus, structural support is the most obvious role, even from the perspective of a single-celled eukaryote. The authors should provide evidence to support their conclusion, or modify it.

*AU: We now mention the possibility that lignin, if present in unicellular organisms, could contribute to the cell wall structure. However, several other compounds have already been shown to fulfill that role in unicellular organisms (silica, cellulose, etc) and lignin does not seem necessary for this purpose. Also, we have not demonstrated that the organisms studied can make lignin, We are suggesting that our evidence supports the production of the H lignin precursor, p-coumaryl alcohol.*

### Reviewer’s report 3

#### Philippe deschamps (nominated by purificacion Lopez-Garcia)

Labeeuw et al. present a genomic survey of the genes coding the enzymes known to be involved in the biosynthesis of lignins, as well as a phylogenetic analysis of three enzymes catalyzing the conversion of *p*-coumaric acid into H monolignol. H monolignol and its substituted forms (G and S) are substrates for the synthesis of lignin in Viridiplantae. The purpose of the article is to determine if monolignols biosynthesis is a specificity of land plants or if there are evidences that the pathway existed before their diversification. In the latter case, did the corresponding pathway emerged or was acquired in the common ancestor of Archaeplastida? An additional goal is to determine if monolignol biosynthesis related genes exist in other eukaryotic lineages, and to understand how they evolved.

From their surveys found in Tables 1 and 2, it is clear that the synthesis of complex lignin is restricted to Streptophyta and that only a portion of the lignin pathway is found in Chlorophyta and Rhodophyta. From the results presented in the same tables, we can observe that there seems to be a correlation between the actual or former existence of a plastid in a lineage and the presence of the 3 enzymes catalyzing the synthesis of H monolignol. From these observations, the authors hypothesize that these three enzymes were inherited by or developed in the common ancestor of Archaeplastida and transmitted to other photosynthetic and related heterotrophic lineages by secondary endosymbioses. To test this hypothesis, they produced a phylogenetic tree for each gene family corresponding to the three enzymes involved in H monolignol synthesis. In their interpretation of these trees, the authors see a strong support for their hypothesis.

If I agree that the presence/absence pattern suggests such a scenario, none of the phylogenetic tree provided show a topology supporting it. First, the monophyly of Archaeplastida is never recovered. I know that this is almost always the case in single gene trees, so this is nonetheless quite possible that the corresponding enzymes were acquired in their common ancestor. Yet, there is an additional problem: as mentioned by the authors, enzymes involved in lignin biosynthesis are often duplicated and sub-functionalized. With this in mind, let's focus on the topology inside the Archaeplastida clade: red algae are not recovered as coherent clades. Moreover, red and green algae possess several copies in each gene family but these copies are shuffled, meaning that they don't follow the expected species phylogeny. The authors use these duplications as a positive argument in favor of a common gene ancestry in Archaeplastida. This conclusion is only possible if paralogous clusters are well defined and observable, but this is not the case. Actually the three phylogenetic trees probably suffer from hidden paralogies, and thus are prone to interpretation errors.

*AU: We have now revised our hypothesis to be less specific in terms of the origins of p-coumaryl alcohol synthesis in eukaryotes. We now propose that it originated either in the shared ancestor of green algae and land plants, or possibly earlier in the archaeplastid lineage. This is in acknowledgement that red algae are not recovered in coherent clades and could have acquired the CAD and CCR genes by LGT. We acknowledge that hidden ancient paralogy is likely present in the phylogeny of the CCR and CAD gene. However, our positive argument in favor of an origin for lignin biosynthesis early in archaeplastid evolution is made mostly using the 4CL gene. This gene does not present rampant paralogy, but only displays it in a single strongly supported clade which is composed solely of red and green algae as well as stramenopiles and a dinoflagellate, which have secondary red plastids. We agree that this is only suggestive of an origin predating the split between red and green algae for the 4CL and not enough to conclude it. We have modified the manuscript to suggest that the pathway could have been assembled by several EGT and LGT events and did not necessarily originate in the ancestor of archaeplastids.*

So, in a context where Archaeplastida are not recovered as monophyletic and display a problematic internal topology, it is impossible to pretend resolving any putative endosymbiotic gene transfer from red algae toward a secondary photosynthetic lineage. Moreover, on each tree, red points are displayed on nodes supposed to support a case of secondary EGT; none of them are compatible with a secondary EGT from a red alga. Based on the observed topology, one could propose with the same (if not more) likelihood that secondary photosynthetic lineages acquired H monolignol synthesis related genes via independent LGT.

*AU: We originally considered a red algal origin to be the most likely explanation for the presence of three putative enzymes of the lignin biosynthesis pathway almost only in organisms known to have secondary red plastids. However, we understand that our evidence for that specific scenario is limited. We have therefore changed our hypothesis substantially to also include the possibilities of both green algal origin (or origin from other ancestral algae) and LGT for all three genes. The red circles on the three trees now simply indicate a putative origin through EGT from either red or green algae or an LGT from a different source.*

Finally, unexpected heterotrophic species are present within Archaeplastida, like Opisthokonts, Amoebozoa and Fungi. This further decreases the ability to correctly interpret the trees.

*AU: We have now performed functional prediction analysis on all 4CL, CCR and CAD homologs found. All homologs without a correct functional prediction were removed from the dataset for phylogenetic analyses. This eliminated a number of non-orthologous homologs from heterotrophic species. There are now very few statistically supported clades containing archaeplastids or secondarily photosynthetic organisms that also contain heterotrophs. In the 4CL tree, the roseobacter clade is the only clade with heterotrophs and archaeplastids, and is a clear case of LGT. In the CCR tree, only two clades contain photosynthetic eukaryotes and heterotrophs. One is a bacterial clade that also includes the diatom P. tricornutum and is a clear case of LGT from bacteria to a eukaryote. The other is a mixed clade with moderate (70%) support including a single red alga (Porphyridium cruentum) as well as bacteria and heterotrophic eukaryotes. This clade is now discussed in the text as illustrating that red algae present a polyphyletic pattern in the CCR and CAD trees and cannot be used to make inferences on the evolution of monolignol biosynthesis. In the CAD tree, a single supported clade shows grouping of a heterotroph (yeast) with a photosynthetic eukaryote (red alga). It is at the very base of the tree and groups paralogs from these organisms that are alcohol dehydrogenases, not cinnamyl alcohol dehydrogenases (very high confidence in functional prediction analysis). These have not been removed from the dataset despite their clear functional prediction as alcohol dehydrogenases, as they both have paralogs in the tree with cinnamyl alcohol dehydrogenase (CAD) function. Besides these few exceptions, the vast majority of nodes grouping archaeaplastids together or archaeplastids with secondarily photosynthetic species is well supported in these trees and contains no heterotrophs. We appreciate the reviewer raising this issue and have now included more detailed explanations of mixed heterotrophs/autotrophs clades, which are in some cases the result of LGT. We have also pinpointed these events in Figures**3**,**4**and**5**.*

Additionally, I think that Figure 6 is highly problematic. First, as I explained above, there is no support for the evolutionary scenario that is depicted. Secondly, such a ?consensus of current phylogenetic analyses of the eukarotic domain? does not exist. The relative position of the SAR, Haptophytes and Cryptophytes is still highly debated and their global monophyly was rejected in recent studies (for example Burki et al. 2012 in PRS.B). Moreover, if the common origin of their plastids from a single red alga seems possible (see Petersen et al. 2014 in GBE), the number of subsequent endosymbioses leading to the diverse secondary red lineages is still undetermined.

*AU: We recognize that the phylogeny of eukaryotic supergroups is controversial in the scientific community. We originally depicted one of the popular theories for this phylogeny: that a single endosymbiotic event was at the origin of the red plastid found in secondarily photosynthetic eukaryotes, which was in agreement with our interpretation of the data. We understand the concerns of the reviewer and have now changed Figure**6**substantially, including uncertainties about the relationship between major eukaryotic groups which are still under discussion in the scientific community. Since we have now changed the article to reflect uncertainty about the origin of putative lignin biosynthesis genes we have identified, the evolutionary events depicted have also been modified, and both LGT and EGT events are now included.*

## Reviewers’ comments – second round of review

### Reviewer’s report 1

#### Mark ragan

The authors have now addressed my concerns.

### Reviewer’s report 2

#### Uri gophna

No further comments.

### Reviewer’s report 3

#### Philippe deschamps (nominated by purificacion Lopez-Garcia)

The revised version of the manuscript presented by Labeeuw et al. takes into account many of the suggestion made by the reviewers. This has clearly enhanced some aspects. For instance, the general comment was that some of the conclusions or “announcements” were hasty considering the data that were actually presented (for example on the ability to synthesize genuine lignin precursors or on evolutionary interpretations of the phylogenetic trees). This specific issue has been corrected.

On the other hand, some decisions made during the revision process, in order to comply with some of the reviewer's comments, have indirectly lowered the rigor of the analysis.

One question raised was, considering the amount of gene paralogies detected in the phylogenetic trees for the four studied gene families, can we be sure that these genes are function homologues? This is an interesting question, especially when the goal is to try to indirectly demonstrate the presence of an active specific pathway in some species. To answer this, the authors have decided to use several tools that compute a prediction of the actual function of a protein based on its primary sequences. The authors mention this in their text, so I won't elaborate on the various issues of these prediction models, and on the fact that they are still not reliable enough to replace proper functional analyses. However, I must insist on a more critical issue. Following their functional predictions, the authors decided to remove from their phylogenetic analyses all proteins that were not predicted as functional homologs of the genes of interest. I quote : “We have now performed functional prediction analysis on all 4CL, CCR and CAD homologs found. All homologs without a correct functional prediction were removed from the dataset for phylogenetic analyses. This eliminated a number of non-orthologous homologs from heterotrophic species. There are now very few statistically supported clades containing archaeplastids or secondarily photosynthetic organisms that also contain heterotrophs (…) ”. What has been done here is absolutely unacceptable. A sequence homolog is not necessarily a functional analog, but the opposite also applies: homologous proteins that differ in their function should not be considered as non-orthologous: orthology is defined by the sequence, not by the function. What the authors did here was to remove these sequences form their analyses and claim that the results are now really more in accordance with their interpretations (for instance: no more heterotrophic species in photosynthetic clades, which I pointed out as a potential flaw in their phylogenetic study). In short: the authors do not realize that this operation is comparable to a falsification.

Anyway, the phylogenetic trees presented in this revised manuscript still don't really support the conclusions. The signal is blurry, sequences are shuffled, probably due to a high degree of hidden paralogy. The authors wrote: “both the CCR and CAD phylogenies likely contain hidden ancestral paralogy and differential loss”. This means that they are OK to explain a patchy distribution by differential loss for some clades. But they also ignore it completely for some other clades where heterothrophic species mix with secondary photosynthetic ones. For these latter, the authors think that photosynthetic species got the genes by E/LGT, and that heterothrophic species are just there, maybe due to isolated LGT or maybe because they are not “real orthologs”. Sorry but, strictly speaking, these mixed clades can also be explained (with the same likelihood) by an ancestral wide distribution followed by differential loss.

My opinion is unchanged: The presence absence distribution suggests that the pathway for *p*-coumaryl alcohol synthesis in eukaryotes could have emerged in Archaeplastida and have been transmitted by endosymbiotic transfers to other lineages. But, on a pure technical point of view, the phylogenetic analyses cannot be objectively used to support this hypothesis. I even consider, due to the kind of changes that were made in these phylogenetic analyses, that this revised version is technically less correct then the original one.

*AU: We understand the reviewer’s concern regarding the removal of homologs lacking functional predictions from the phylogenies of 4CL, CCR and CAD homologs. We have added these back to the phylogenies and these sequences did not significantly change the clades on which we based our conclusions, key clusters remaining well supported statistically. We agree with the reviewer that the trees generally have a weakly supported backbone and that the CAD and CCR trees are likely affected by some degree of ancestral paralogy. However, our conclusions are based on nodes that are well supported statistically (which is clearly indicated in the figures). Also, the phylogenetic pattern in which secondarily photosynthetic organisms from widely divergent taxonomic groups strongly cluster together is repeated in the trees of all three genes. We consider it highly unlikely for the observed pattern to be the result of ancient paralogy and differential losses happening in the same complex way for three separate genes. In our opinion, it is much more likely the result of a common origin, as suggested in our hypothesis of EGT between an ancestral archaeplastid and the ancestors of secondarily photosynthetic organisms. The reviewer also mentions the alternate possibility that p-coumaryl alcohol biosynthesis gene homologs were present in an ancient eukaryotic ancestor and lost in numerous lineages. We have now added a few statements in the text presenting this alternate hypothesis.*

## Abbreviations

4CL: 4-coumarate:CoA ligase
C3H: *p*-coumarate 3-hydroxylase
EGT: Endosymbiotic gene transfer
C4H: Cinnamate 4-hydroxylase
CAD: Cinnamyl alcohol dehydrogenase
CCoAMT: Caffeoyl-CoA *O*-methyltransferase
CCR: Cinnamoyl-CoA reductase
COMT: Caffeic acid *O*-methyltransferase
CSE: Caffeoyl shikimate esterase
F5H: Ferulate 5-hydroxylase
HCT: Hydroxycinnamoyl-CoA
LAC: Laccase
LGT: Lateral gene transfer
PAL: Phenylalanine ammonia-lyase
PER: Peroxidase
smF5H: *Selaginella moelledorfii* F5H (smF5H)
